# Impact of neoadjuvant chemotherapy on local breast microbiota and macrophages in ER-positive breast cancer with links to treatment response

**DOI:** 10.1038/s41523-026-01004-0

**Published:** 2026-07-06

**Authors:** Sabine Dieleman, Carlijn Bruggeling, Phuc Dat Le, Lars E. Hillege, Catarina Correia Tavares Bernardino, Janine Ziemons, Renske Cremers, Arnaud Jacquerie, Alem Gusinac, Thomas H. A. Ederveen, Kiek Verrijp, John Penders, Anne-Marie Vos, Loes F. S. Kooreman, Heike I. Grabsch, Marjolein L. Smidt, Annemarie Boleij

**Affiliations:** 1 Department of Surgery, GROW - School for Oncology and Reproduction, Maastricht University Medical Center+, Maastricht, The Netherlands; 2 Department of Pathology, GROW - School for Oncology and Reproduction, Maastricht University Medical Center+, Maastricht, The Netherlands; 3 Department of Pathology, Radboud University Medical Center (Radboudumc), Nijmegen, The Netherlands; 4 Department of Medical Biosciences, Radboud University Medical Center (Radboudumc), Nijmegen, The Netherlands; 5 Department of Medical Microbiology, Infectious Diseases, and Infection Prevention, Maastricht University Medical Center+, Maastricht, The Netherlands; 6 NUTRIM - School of Nutrition and Translational Research in Metabolism, Maastricht University, Maastricht, The Netherlands; 7 Euregional Microbiome Center, Maastricht, The Netherlands; 8 Pathology and Data Analytics, Leeds Institute of Medical Research at St. James’s, University of Leeds, Leeds, UK

**Keywords:** Cancer, Microbiology, Oncology

## Abstract

Gut microbiota can modulate the tumor microenvironment and influence therapeutic outcomes in breast cancer. We hypothesized that similar interactions occur between the breast microbiota and tumor microenvironment. This exploratory study evaluated whether in situ microbial and macrophage densities differ between good and poor neoadjuvant chemotherapy responders and compared microbial density and composition post treatment with surgery-only controls. In 20 postmenopausal estrogen receptor-positive breast cancer patients, bacterial density was quantified by fluorescence in situ hybridization and macrophages density by multiplexed immunofluorescence in pre and post treatment tissue. Bacterial composition was analyzed by 16S rRNA sequencing of post-treatment resection specimens. Nine surgery-only patients served as controls. Neoadjuvant chemotherapy significantly reduced CD68 macrophage density, but not CD163 macrophages or bacterial density. Macrophages and bacterial densities were not correlated, and bacterial densities did not differ between good and poor responders. Higher pretreatment CD68 and CD163 macrophage densities predicted good response. Post treatment resections showed reduced bacterial density and alpha-diversity compared to surgery-only controls. These findings suggest that neoadjuvant chemotherapy affects local breast microbiota and macrophages, with macrophages as promising biomarkers to predict treatment response. The interaction between breast microbiota and local immunity deserves more attention in future research.

**Figure:**
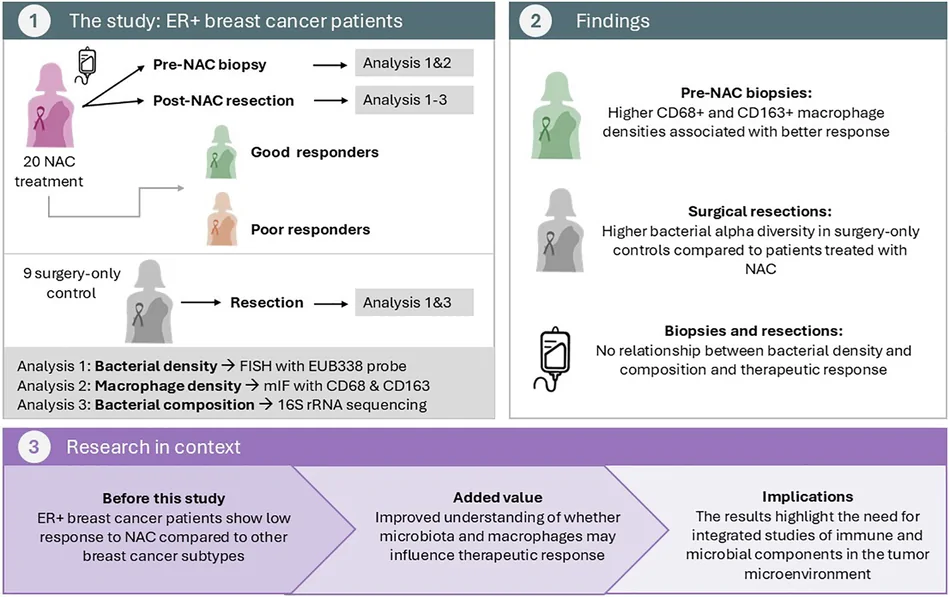

## Introduction

Neoadjuvant chemotherapy (NAC) is administered to breast cancer patients to shrink the tumor, enabling less extensive surgery of the breast and/or axilla, and in selected cases, allowing omission of surgery. Pathological complete response (pCR), defined as the absence of residual disease in both the breast tissue and the regional lymph nodes in the surgical specimen after NAC, is associated with improved long-term outcomes^1^. However, patients with estrogen receptor (ER)-positive breast cancer show less response to NAC compared to those with ER-negative breast cancer^2^. A better understanding of the mechanisms underlying poor response to NAC in ER-positive breast cancer is essential for improving patient stratification, enabling the identification of those most likely to benefit from treatment, and ultimately improving therapeutic efficacy.

With robust biomarkers missing in predicting response to anti-cancer therapy, the microbiome is increasingly recognized as a factor possibly influencing therapeutic efficacy. Gut microbiota influences the effectiveness and toxicity of chemotherapy in cancer, via mechanisms including immunomodulation, microbial metabolism and enzymatic degradation^3^. Antibiotic-induced disruption of gut microbiota impaired the function of tumor-infiltrating myeloid-derived cells, weakening the tumor response to both immunotherapy and cytotoxic chemotherapy in mouse models of lymphoma, colon cancer and melanoma^4^. In an ER-positive breast cancer mouse model, antibiotic treatment resulted in a substantial increase of myeloid cells in the tumor microenvironment (TME), which expressed high levels of the inflammatory mediators arginase-1 and IL-6^5^. Together, these findings suggest that interactions between the microbiota and macrophages may shape the TME and influence therapy outcomes.

Recent studies suggest that solid tumors, including breast cancer, harbor their own local microbiota^6^. The breast-resident microbiota differs in patients with and without breast cancer^7–9^. The association between breast cancer microbiota and the tumor immune microenvironment has not been studied in detail. In a murine model, the presence of intratumoral *Fusobacterium nucleatum* was associated with local breast cancer progression and development of lung metastasis as well as with a decrease in intratumoral CD4+ and CD8+ T cells^10^. In a human breast cancer study investigating the relationship between breast microbial and immune cell signatures, network analysis revealed that the microbiota–immune network in tumor tissue was less connected and lacked several bacterial “hub” taxa, that were present in healthy tissue^11^. In the same study, the Toll-like receptor (TLR) signaling pathway was among the most altered immune pathways, showing downregulation of TLR4 and upregulation of the downstream genes MYD88 and IRAK1 in tumor tissue compared to healthy breast tissue^11^. This suggests that microbial sensing through TLR4 is diminished in tumor tissue, while downstream signaling remains activated, indicating a potentially dysregulated microbiome–immune interaction in the TME.

Available evidence suggests that tumor-associated macrophages (TAMs) are relevant to NAC response in breast cancer, but their role appears to depend on macrophage phenotype and tumor subtype. In triple negative breast cancer, higher infiltration by CD163-positive M2-like macrophages has been associated with incomplete response and lower pCR rates^12^. By contrast, relatively little is known about ER-positive breast cancer. Gene expression-based analyzes suggest that a higher M1 macrophage signature is associated with higher pCR rates and better survival in ER-positive disease, whereas higher M0 macrophage fractions have been linked to worse outcomes; no clear association with M2 macrophages was observed^13^.

We hypothesized that microbiota and macrophage densities in pre-NAC biopsies are positively associated and differ between good and poor responders to NAC. We further hypothesized that NAC significantly reduces both bacterial and macrophage populations. We specifically focus on total tumor-infiltrating macrophages using a pan-macrophage marker, as well as M2-like macrophages, because tumor-associated microbiota potentially interact with the immune microenvironment and may drive M2 remodeling via microbial metabolites or TLR-ligands^14^. The objectives of this study were to (1) examine the association between NAC response and bacterial and macrophage densities in pre-treatment biopsies and matched post-treatment resection specimens of patients with ER-positive breast cancer, (2) assess the impact of NAC on bacterial density and composition, as well as on the density of tumor-infiltrating macrophages, by comparing pre-treatment biopsies with matched post-treatment resection specimens, and (3) compare microbial composition and density in post-NAC resection specimens with those from patients who underwent surgery alone.

## Results

### Patient and tumor characteristics

The median (range) age at diagnosis of the 20 patients who received NAC was 58 (55–62) years, and of the 9 surgery-only control patients 63 (59–68) years. Half of the NAC-patients (*n* = 10) were classified as good responders, of which four patients had a pCR and six were classified as EUSOMA 2 (i) response (Table 1, and Supplementary Table 1). No significant differences in clinicopathological characteristics were observed between good and poor responders (Table 2, and Supplementary Table 2). Tumors in NAC-treated patients had a significantly larger clinical tumor size at the time of diagnosis (prior to NAC and prior to surgery) compared to the surgery-only control group (*p* = 0.007) (Table 2).

**Table 1:** Clinicopathological details of patient cohort

	NAC patients *N* = 20, *n* (%)	Surgery only patients (controls) *N* = 9, *n* (%)	*p*-value
Age at diagnosis in years (median, IQR)	58 [55–62]	63 [59–68]	*P* = 0.068
**Clinical tumor stage**			
cT1	4 (20%)	7 (77.8%)	*P* = 0.007
cT2	12 (60%)	2 (22.2%)	
cT3	2 (10%)	0	
cT4	2 (10%)	0	
**Pathological tumor stage**			
pT0	3 (15%)	0	*P* = 0.127
pT1	10 (50%)	3 (33.3%)	
pT2	7 (35%)	6 (66.7%)	
**Pathological tumor grade (biopsy)**			
Grade 1	5 (15%)	2 (22.2%)	*P* = 0.627
Grade 2	11 (55%)	4 (44.4%)	
Grade 3	4 (25%)	3 (33.3%)	
**Focality**			
Unifocal	16 (80%)	6 (66,7%)	*P* = 0.594
Multifocal	0	3 (33.3%)	
No rest tumor (pCR)	4 (20%)	NA	
**Tumor type**			
NST	18 (90%)	4 (44.4%)	*P* = 0.198
Lobular	2 (10%)	2 (22.2%)	
Mucinous	0	1 (11.1%)	
Missing	0	2 (22.2%)	
**PR status**			
Positive	10 (50%)	4 (44.4%)	*P* = 0.835
Negative	10 (50%)	5 (55.5%)	
**Pathological response**			
EUSOMA 1 (pCR)	4 (20%)	NA	NA
EUSOMA 2 (i)	6 (30%)		
EUSOMA 2 (ii)	4 (20%)		
EUSOMA 2 (iii)	6 (30%)		
**Response groups**			
Good responder	10 (50%)	NA	NA
Poor responder	10 (50%)		

IQR = interquartile range, NST = no special type, pCR = pathological complete response, good responder = EUSOMA 1 or EUSOMA 2(i), poor responder = EUSOMA 2(ii) or 2(iii).

**Table 2:** EUSOMA classes and responder groups

EUSOMA class	Description	Responder group
EUSOMA 1 (i)	No residual carcinoma (complete response)	Good responders
EUSOMA 1 (ii)	No residual invasive carcinoma but presence of carcinoma in situ (DCIS)	
EUSOMA 2 (i)	Partial response; minimal residual invasive carcinoma	
EUSOMA 2 (ii)	Partial response; 10–50% of vital tumor remaining in the tumor bed	Poor responders
EUSOMA 2 (iii)	Partial response; >50% of tumor remaining	
EUSOMA 3	No evidence of response to therapy	

### Bacterial and macrophage density pre- and post-NAC

**Fig. 1:**
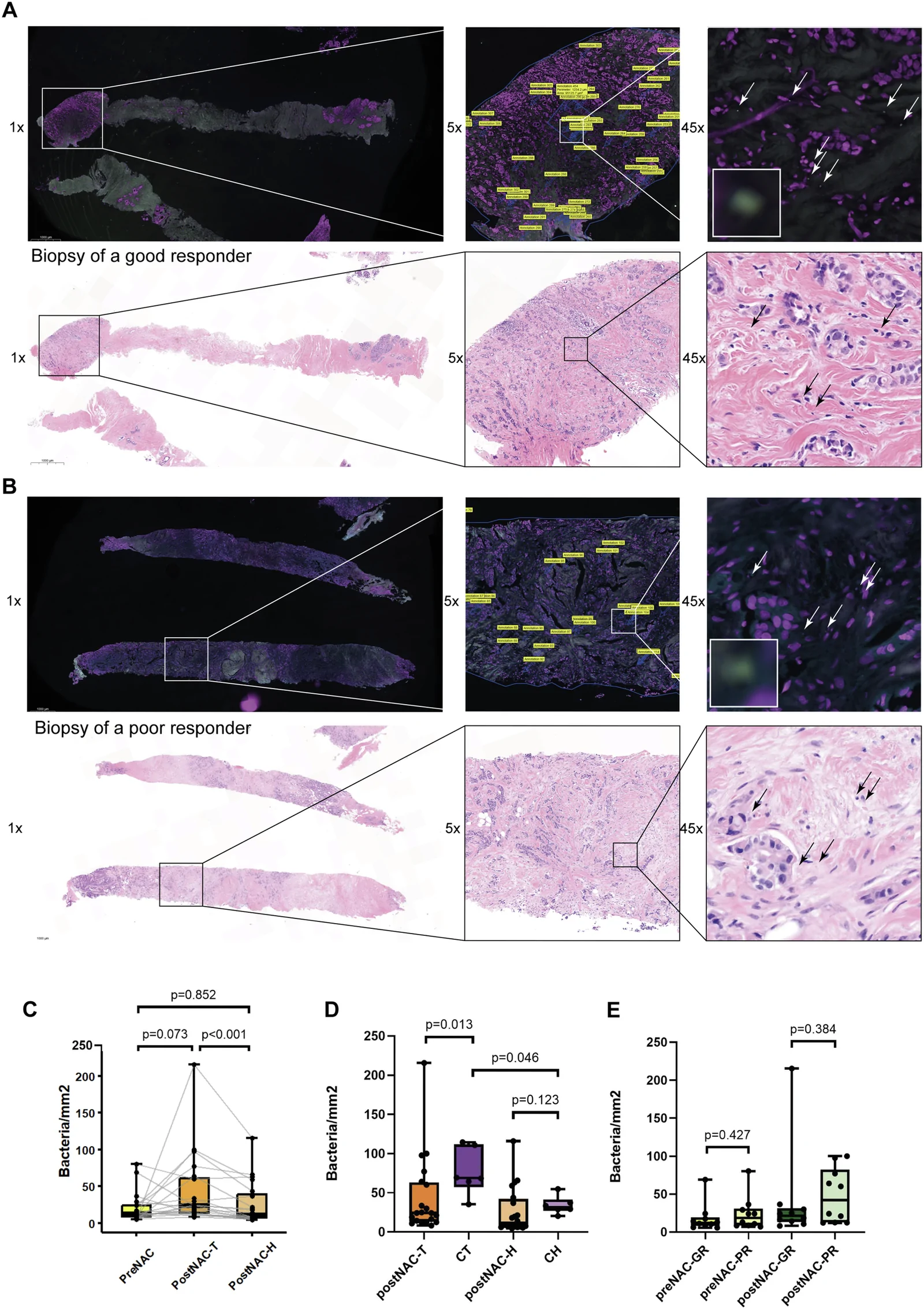
Bacterial detection and quantification in biopsies and resections. FISH and corresponding H&E images of pre-NAC biopsies of a good responder (**A**) and a poor responder (**B**) patient. In the 5x zoom the manual annotations of bacteria are shown within the tumor region of the biopsy, where each yellow label marks an annotation. In the 45x zoom images bacteria are indicated with white arrows. The inlay shows a 300x zoom of a positive signal visible as green due to the dual label of the probe in the cy3(blue-label) and atto-633(yellow-label). The bacteria are detected within the stroma area and measure around 2 µm in length. The boxplot figures presented in **C**, **D** and **E** illustrates bacterial densities in breast cancer tissue. **C** Bacterial counts in patients treated with neoadjuvant chemotherapy (NAC; *N* = 20), comparing pre-treatment biopsies (PreNAC) with post-treatment tumor areas (postNAC-T) and adjacent healthy breast tissue (postNAC-H). **D** Comparison of bacterial densities in postNAC-T and postNAC-H (*N* = 20) with those from untreated control patients (*N* = 6), specifically control tumor tissue (CT) and control healthy tissue (CH). **E** Bacterial densities in PreNAC biopsies and postNAC-T tissue, stratified according to pathological response to NAC, distinguishing between good responders (GR; PreNAC-GR, PostNAC-GR; *N* = 10) and poor responders (PR; PreNAC-PR, PostNAC-PR; *N* = 10).

**Fig. 2:**
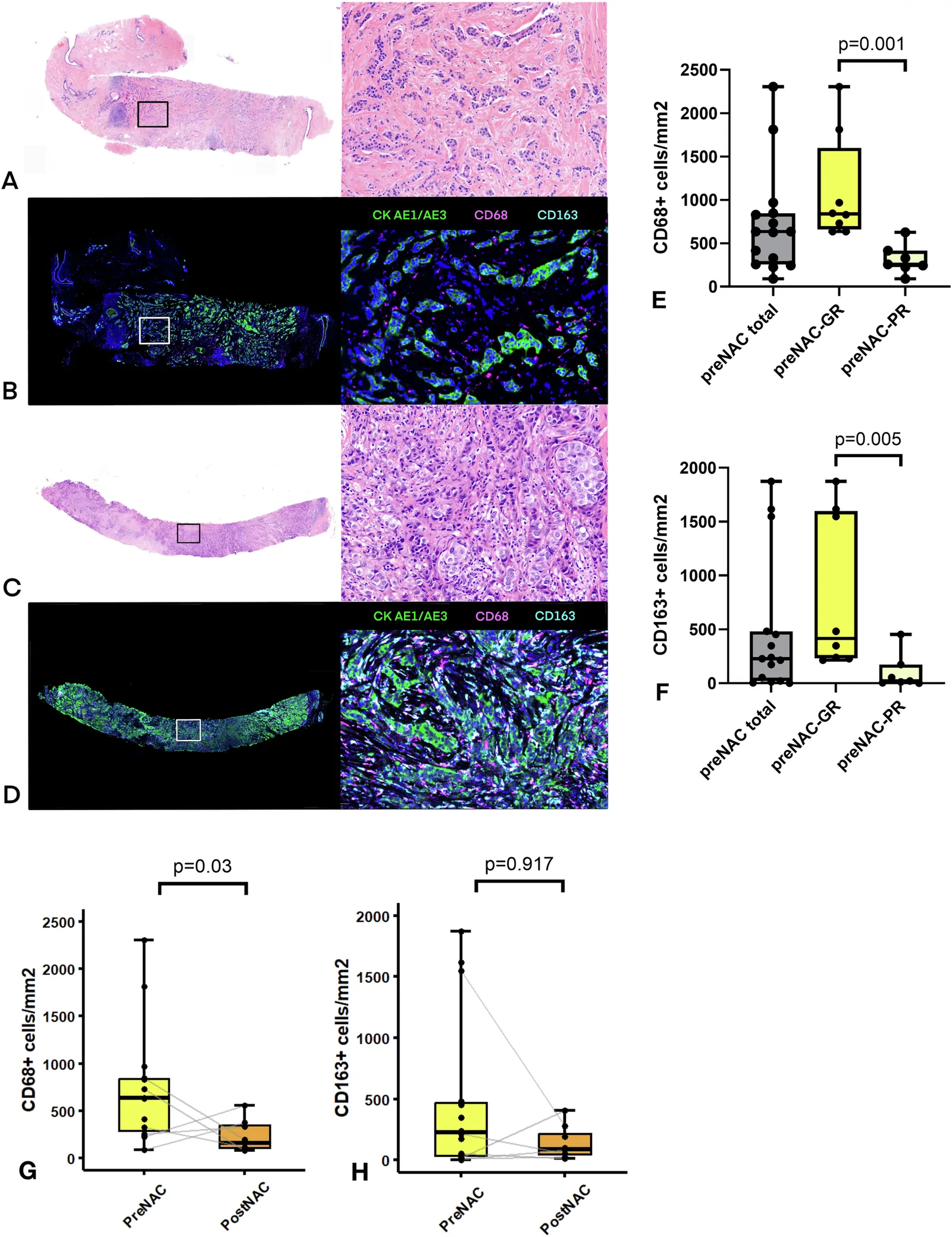
Immunofluorescent quantification of macrophages in pre-neoadjuvant chemotherapy (pre-NAC) biopsies from poor and good responders. **A**, **B** Representative hematoxylin and eosin (H&E) staining and multiplex immunofluorescence (mIF) imaging of a pre-NAC biopsy from a poor responder breast cancer patient, while **C**, **D** show corresponding images from a good responder patient. All left panels are at 2.5x magnification and all right panels are at 20x magnification. Macrophage infiltration was quantified and is presented in **E** and **F** as boxplots depicting cell counts per mm². **E** The density of CD68⁺ macrophages, and **F** shows the density of CD163⁺ macrophages. In both panels, total macrophage counts across all pre-NAC samples (*N* = 16) are displayed alongside subgroup-specific counts for good (*N* = 8) and poor responders (*N* = 8). **G**, **H** A decrease in macrophage density following NAC (preNAC *N* = 16, PostNAC *N* = 6) for both CD68⁺ and CD163⁺ macrophages. However, this reduction is only statistically significant for CD68⁺ macrophages (*p* = 0.03) (**G**), while the decrease in CD163⁺ macrophages is not significant (*p* = 0.917) (**H**).

CD68 was used as a pan-macrophage marker reflecting overall macrophage infiltration, whereas CD163 was used as a marker of M2-like macrophages, which are commonly associated with immunosuppressive and tumor-promoting functions. Bacteria were observed in diverse tissue compartments, including tumor stroma, immune cells, and epithelial cells, without a consistent localization pattern, meaning they were not preferentially or uniformly confined to a specific tissue compartment across samples. Bacterial density was assessed by (kingdom) Bacteria fluorescence in situ hybridization (FISH) and quantified as the number of bacteria per mm² (Fig. 1A, B). In NAC-treated patients, bacterial density within tumor tissue did not differ between pre-NAC biopsies and matched post-NAC resections (*p* = 0.073; Fig. 1C). In contrast, CD68+ macrophage density decreased following NAC (*p* = 0.03), while there was no difference in CD163⁺ macrophage density between pre- and post-NAC samples (*p* = 0.917) (Fig. 2G, H). No correlation was found between bacterial density and macrophage densities in either pre- or post-NAC samples (Supplementary Fig. 1).

### Impact of NAC on bacterial density compared to surgery only controls

To further assess the impact of NAC treatment on the breast cancer microbiota, bacterial density was compared between NAC-treated patients and surgery-only control patients. Bacterial density was significantly lower in tumor tissue from NAC-treated patients compared to surgery-only controls (*p* = 0.013, Fig. 1D), while no differences were observed in adjacent non-tumor tissue. In both NAC-treated and surgery-only control patients, bacterial density was higher in tumor areas than in adjacent normal tissue (Fig. 1C).

### Comparison of microbiota and macrophage density in good versus poor responders

Bacterial density did not differ between good and poor responders in the pre-NAC biopsies (*p* = 0.427) or in the post-NAC resections in tumor areas (*p* = 0.384) (Supplementary Fig. 2). In contrast, higher densities of CD68+ and CD163+ macrophages were seen in pre-NAC biopsies of good responders compared to poor responders (*p* = 0.001 and *p* = 0.005, respectively). Representative H&E and mIF images of pre-NAC biopsies of a poor and a good responder are shown in Fig. 2A–D.

### Impact of NAC on bacterial composition compared to surgery only controls

Taxonomic profiling identified Proteobacteria, Actinobacteria, and Firmicutes as the dominant phyla across all samples. *Pseudomonas* was consistently overrepresented, including in tissue-free paraffin control samples processed in parallel, suggesting potential contamination introduced during tissue processing. A phylogenetic analysis of all *Pseudomonas* amplicon sequence variants (ASVs) in breast tissue samples and paraffin controls (Supplementary Figs. 3 and 4) showed that ASVs 37-50 were exclusive to paraffin controls, while ASV 27 was the predominant variant in tissue samples from both collection sites. Notably, Catharina Hospital and Elkerliek Hospital share one diagnostic laboratory, while Maastricht University Medical Centre+ processes specimens in its own laboratory.

To assess whether the observed *Pseudomonas* abundance in sequencing data reflected bacterial load, we performed immunohistochemistry (IHC) using an antibody against *Pseudomonas*, and compared bacterial counts per mm² to (kingdom) Bacterial signals detected by FISH in pre-NAC biopsies (Supplementary Fig. 5). IHC revealed a significantly lower abundance of *Pseudomonas* (2.5 bacteria /mm^2^, SD1.7) compared to (kingdom) Bacteria FISH (17.2/mm^2^, SD9.8; *p* < 0.001). A similar trend was observed in post-NAC resections with *Pseudomonas* counts lower than (kingdom) Bacteria FISH, though not statistically significant (*p* = 0.056).

**Fig. 3:**
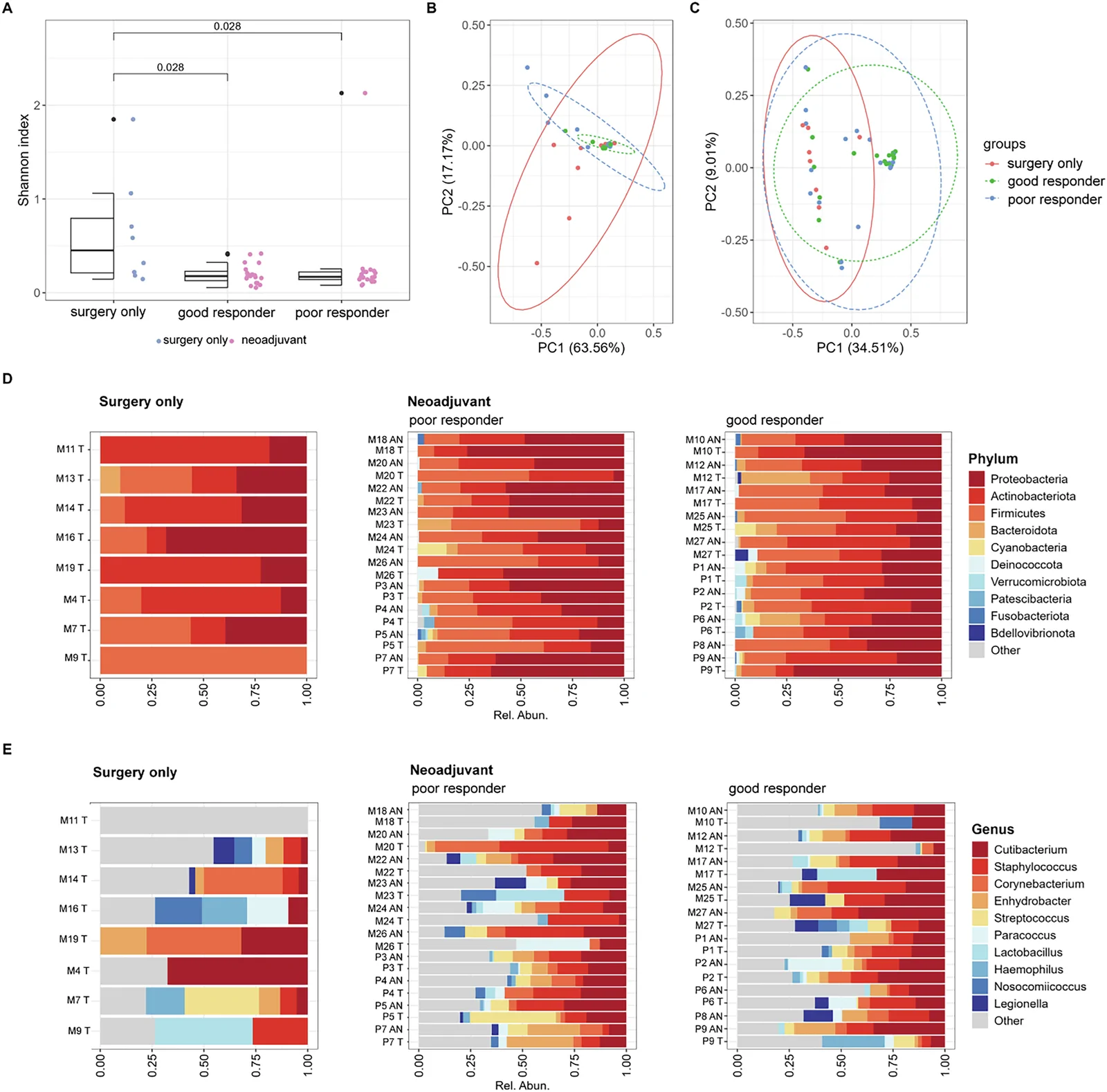
Microbial diversity and composition in breast cancer patients receiving neoadjuvant chemotherapy versus surgery-only controls. **A** Alpha diversity computed by the Shannon index for surgery-only controls (*N* = 8) and patients undergoing neoadjuvant chemotherapy (*N* = 20). The significance based on the Mann–Whitney test shows a significant difference with neoadjuvant treatment versus control. The Principal Coordinate Analysis (PCoA) based on the weighted (**B**) and unweighted (**C**) UniFrac distances. For both the weighted (*P* = 0.012) as unweighted (*P* = 0.003) UniFrac a significant difference has been observed for the good responders (*N* = 10) and surgery-only controls (*N* = 8). There was no significant distinction between poor and good responders (unweighted *P* = 0.828), nor between poor responders and surgery-only control patients (unweighted *P* = 0.054). **D** Overview of the relative abundance of Phylum for the different groups. **E** Overview of the relative abundance of Genera for the different groups.

Given the discrepancy between sequencing and IHC data for *Pseudomonas*, we kept the *Pseudomonas* during the alpha- and beta-diversity analysis since they were equally prevalent in all samples (Fig. 3A–C), while excluding them for better visualization of the other bacteria (Fig. 3D, E). The removal of 23 ASVs belonging to the *Pseudomonas* resulted in the removal of 1,081,197 out of 1,139,957 reads (Supplementary Table 3). Commonly detected genera included *Cutibacterium, Staphylococcus, Corynebacterium, Enhydrobacter, Streptococcus, Paracoccus, Lactobacillus, Haemophilus, Nosomilicoccus*, and *Legionella* in all samples (Fig. 3E). Alpha diversity, measured by the Shannon index, was significantly reduced in NAC-treated patients compared to surgery-only controls (Fig. 3A). Principal Coordinate Analysis (PCoA) of beta diversity revealed differences in microbial communities between good responders and surgery-only control patients (Fig. 3B, C), indicating distinct overall microbial community structure. There was no significant distinction between poor and good responders, nor between poor responders and surgery-only control patients.

## Discussion

In ER-positive breast cancer, the response to NAC is generally low compared to other breast cancer subtypes, and identification of factors that influence treatment efficacy could provide new insights into poor therapeutic response and support the development of more tailored treatment strategies. Prior studies have suggested that response might be dependent upon the composition of the immune microenvironment within the primary breast cancer^13,15–18^. The role of breast microbiota in shaping the local immune milieu, how these are impacted by chemotherapy, and how they relate to therapeutic response has not yet been investigated. While bacterial density and diversity did not correlate with response to NAC, this study revealed that high macrophage density (CD68+ and CD163+) in pre-NAC biopsies was significantly associated with better treatment response.

This exploratory study could not demonstrate that NAC significantly altered bacterial density within the tumor. Bacteria remained more abundant in tumor tissue compared to adjacent non-malignant tissue across all patients. Although our study size is small, this aligns with previous work by Nejman et al., who demonstrated that breast tumors harbor a richer bacterial community than surrounding tissue^6^. The persistence of bacteria within the TME, despite systemic chemotherapy, supports the hypothesis that these microorganisms may influence local immune processes as was shown in a breast cancer mouse model. In this model, tumor infiltration with *F. nucleatum* resulted in local tumor progression and metastasis by suppression of CD4⁺ and CD8⁺ T lymphocyte accumulation in the TME^10^. However, we found no association between bacterial density and treatment response in this exploratory analysis.

TAMs are a heterogeneous population broadly categorized into pro-inflammatory, anti-tumorigenic M1-like macrophages and immunosuppressive, pro-tumorigenic M2-like macrophages^19^. Because tumor-associated microbiota potentially interact with the immune microenvironment and may drive M2 remodeling via microbial metabolites or TLR-ligands^14^, we used CD68, a commonly used pan-macrophage marker detecting both M1- and M2-like populations, and CD163, a marker for M2-like macrophages^19^. In our study, macrophage infiltration, specifically CD68⁺ and CD163⁺ cells, emerged as a promising indicator of therapeutic response, with higher densities in pre-NAC biopsies significantly associated with better outcomes. These findings are consistent with previous gene expression studies linking M1-like macrophages to favorable chemotherapy response in ER-positive breast tumors^13,15,20^. However, the literature describes conflicting roles for TAMs, likely reflecting heterogeneity in macrophage subtypes^21^ examined as well as differences in experimental methodologies. TAMs, and not T cells, are the most predominant immune cells infiltrating the tumor bed in ER+ breast cancer and differ between ductal and lobular subtypes in their transcription. In a recent study, macrophage infiltration into the tumor bed and their interaction with T cells specifically related to longer disease-free survival in ductal carcinomas but not in lobular breast cancer. This functional difference may be tumor-driven, with lobular carcinomas having a greater ability to polarize monocytes into M2-like macrophages compared to ductal carcinomas^22^. In a study across breast cancer subtypes, including ER-positive breast cancer, immunohistochemically assessed CD163⁺ macrophages were significantly associated with pCR, whereas CD68⁺ macrophages were not. Conversely, single-cell RNA sequencing in ER-positive breast cancer found higher macrophage abundance in both pre- and post-NAC specimens of poor responders compared to good responders^23^.

Although we hypothesized that the microbiota might modulate macrophage infiltration or polarization, we did not observe a direct correlation between bacterial and macrophage density. Nevertheless, this interaction remains biologically plausible and may be better investigated in a pre-clinical setting^24^. Microbial components such as lipopolysaccharides can engage TLR on immune cells and stimulate cytokine production, potentially affecting immune recruitment and function^25^. In a colorectal cancer murine model, for example, *F. nucleatum*, a Gram-negative bacterium associated with CRC, has been shown to drive M2 macrophage polarization^26^. The absence of a detectable association in our cohort may be explained by several factors. First, the absence of spatial resolution of bulk tissue analysis may obscure localized interactions between specific microbial populations and immune cells. Second, the diversity and abundance of the microbiota in breast cancer may be lower than in gastrointestinal cancers, potentially limiting their immunomodulatory impact. Importantly, the investigation of this relation pre-NAC was hampered by the amount of tissue available for analysis, and is a challenge to investigate such associations in a neoadjuvant setting.

Differences in breast microbiota composition have been observed between breast cancer, adjacent breast tissue surrounding breast cancer, and non-malignant breast tissue^6,27^.

The effect of NAC on the bacterial microenvironment in breast cancer remains poorly characterized, with Chiba et al. currently representing the sole study to have addressed this topic^28^. We found decreased alpha-diversity in patients treated with NAC compared to patients treated with surgery alone, independent of response. This is in line with the findings of Chiba et al., demonstrating reduced alpha-diversity in post-treatment resection specimens of 15 NAC-treated breast cancer patients compared to 18 patients treated with surgery alone, though the total bacterial presence was not altered^28^. However, their analysis did not examine the relationship between breast microbiota composition and therapeutic response. We compared treated with surgery only control patients to explore whether NAC might affect bacterial composition, which in turn could influence the TME and treatment outcomes. Because of the small size of diagnostic biopsies, microbial composition before NAC could not be assessed in patients treated with NAC. Nonetheless, our findings in combination with the findings of Chiba et al. suggest that NAC has an effect on breast microbiota, though it needs further investigation to determine whether breast microbiota composition influences treatment response.

Our 16S rRNA sequencing data identified Pseudomonas as the most abundant genus in breast cancer tissue following NAC. Given the different diagnostic workflows at the two processing sites, the detection of ASV27 (classified as *Pseudomonas*) in samples from both locations suggests it is unlikely to originate from paraffin contamination. Instead, it may represent a common contaminant introduced during sample processing or a genuine resident of the breast tissue microbiome. However, the overrepresentation observed in sequencing data was not reflected in our IHC analysis, which detected only limited *Pseudomonas* presence in tumor tissue. Chiba et al. previously reported increased *Pseudomonas* abundance after chemotherapy and enrichment in tumor compared to adjacent normal tissue^28^, and other studies have similarly identified *Pseudomonas* as enriched in breast cancer tissue^11^. While our microbial composition data align with these reports, the discrepancy with IHC underscores the importance of complementary histological validation to accurately assess microbial load and spatial distribution in the TME.

Strengths of this study include the homogeneous patient population, consistent treatment protocols, and the combined use of in situ bacterial visualization by FISH and molecular profiling by 16S rRNA sequencing. The exclusion of recent antibiotic use minimized confounding effects on microbiota composition. However, several limitations should be acknowledged. This was an exploratory study with a relatively small sample size, which may have limited our ability to detect subtle associations between microbiota and macrophages and treatment response. Due to the small sample size, we have limited our analysis to univariable comparisons. Stratified analysis on subgroups, e.g., histotypes (lobular vs ductal) or multivariable regression analysis including age, tumor size, and stage, was not feasible. The consequence is some level of heterogeneity within our NAC and surgery-only groups that we could not take into account. The use of formalin-fixed paraffin-embedded (FFPE) tissue for 16S rRNA sequencing, while pragmatic, is more prone to contamination than fresh-frozen tissue. Although we accounted for this by excluding outer paraffin sections and implementing stringent filtering, the low biomass of breast samples remains a challenge due to potential background noise. Additionally, some patients were excluded from analysis due to insufficient DNA quality, which further limited the sample size. Bacterial composition could not be assessed in pre-NAC biopsies due to limited material availability, precluding comparative analyzes for pre-NAC versus post-NAC bacterial composition. Future studies with larger, prospectively collected cohorts are needed to robustly explore the relationship between macrophages and the tumor-associated microbiota. Moreover, the role of the gut microbiome, which may influence tissue-resident macrophages prior to therapy, warrants further investigation in the context of breast cancer.

In conclusion, we demonstrated that bacterial density remained high in tumor tissue both before and after NAC, and that tumors were enriched in bacteria compared to adjacent non-malignant tissue. Macrophages emerged as a promising indicator of therapeutic response, as higher pre-treatment macrophage densities were associated with improved NAC outcomes, suggesting potential value for predicting treatment response in ER-positive breast cancer. Although our data do not support a direct relationship between intra-tumoral bacteria and immune cell density, the persistent presence of bacteria in ER-positive breast cancer highlights the need for further investigation into how the tumor-associated microbiome may influence the immune microenvironment and, ultimately, therapeutic response.

## Methods

### Study design and patient selection

This study was part of a prospective cohort study conducted in the Netherlands between 2017 and 2020. For this prospective study, eligible participants were postmenopausal women diagnosed with ER-positive, HER2-negative breast cancer^29^. From this prospective cohort, twenty patients receiving NAC were included in this exploratory study as the experimental group, including Catharina Hospital, Elkerliek Hospital and Maastricht University Medical Centre+. Nine chemotherapy-naïve patients treated with surgery only from Maastricht University Medical Centre+ served as controls to compare the impact of NAC on the microbiome, because it was impossible to use pre-NAC biopsies for this purpose. Exclusion criteria included systemic therapy within 1 month before inclusion, oral antibiotic use within the past 3 months, and distant metastatic disease. Due to technical challenges, tissue was occasionally lost during sample processing, particularly in fragile and fatty tissue sections, resulting in different sample numbers across analyses. The exact *N* for each analyzes is summarized in Supplementary Fig. 6. Ethical approval for this study was obtained by the Medical Ethics Committee azM/UM (METC 2021-2603-A-2). The present study was based on data from a prospective cohort^29^, for which participants had previously provided informed consent. The requirement to obtain renewed informed consent for the present analysis was waived by the ethics committee.

### Neoadjuvant chemotherapy regimen and tissue collection

All NAC patients received four cycles of Adriamycin (Doxorubicin; 60 mg/m²) and cyclophosphamide (600 mg/m²), followed by four cycles of docetaxel (100 mg/m²), every 3 weeks. FFPE core biopsy tissue was collected pre-NAC, and surgical resections were collected post-NAC. From surgery-only patients, surgical resection specimens were collected. For the tumor paraffin block, the tissue section with the highest proportion of tumor was selected, while for the adjacent non-malignant tissue, a block located near the tumor but free of malignant cells was chosen. Tumor tissue was annotated by a specialized breast pathologist on digital hematoxylin-eosin-stained slides. In patients with pCR (*n* = 4), scar tissue instead of tumor tissue was analyzed. This approach allowed inclusion of post-NAC tissue from all patients, capturing the full spectrum of treatment response while ensuring anatomical comparability by sampling from the original tumor bed.

### Assessment of treatment response

Treatment response was evaluated by a specialized breast pathologist using the EUSOMA classification^30^. Response was classified according to EUSOMA criteria, as this approach captures the spectrum of pathological response beyond the binary pCR definition by incorporating residual disease. Good responders were defined as patients with EUSOMA 1 and EUSOMA 2 (i), and poor responders as patients with EUSOMA 2 (ii), EUSOMA 2 (iii) and EUSOMA 3 (Table 2).

### Fluorescence in situ hybridization (FISH) to establish bacterial presence

FISH was used to visualize bacterial presence using the universal (kingdom) Bacteria probe EUB338 (5′-GCT GCC TCC CGT AGG AGT-3′) labeled with Cy3-ATTO633 (Biolegio)^31^. Four-micrometer FFPE tissue sections were cut aseptically, deparaffinized by submersion in 100% Xylene twice and 99.5% Ethanol (EtOH) twice for 5 min each. Next, the tissue sections were hybridized overnight at 46 °C with EUB338 (5′-GCT GCC TCC CGT AGG AGT-3′) labeled with Cy3-ATTO633 (Biolegio) in 20 mM Tris-HCl, pH 8.0, 0.9 M NaCl, and 0.01% sodium dodecyl sulfate. Unbound probes were removed by washing 3 times for 5 min in 48 °C washing buffer containing 20 mM Tris-HCl and 0.9 M NaCl, 1 time for 1 min in cold sterile water and 1 time for 1 min in room temperature phosphate-buffered saline. Slides were mounted with 4′,6-diamidino-2-phenylindole (DAPI) Fluoromount-G (SouthernBiotech, 0100-20) and scanned at 20× magnification using a fluorescence scanner (Pannoramic MIDI II, Sysmex) with exposure time of 20,000 ms in the blue (380–598 nm), green (488–600 nm), red (550–650 nm), and far-red (650–750 nm) channels. NonEUB338, with the reverse complementary sequence to EUB338, labeled with Cy3-ATTO633, was used on every tissue slide as a negative control to account for nonspecific binding (Biolegio)^31^.

### Bacterial quantification

Bacteria were visualized in CaseViewer (3DHISTECH Kft, Budapest, Hungary) at 90× magnification using the images from the red (Cy3) and far-red (ATTO633) channels. Green autofluorescence and negative control staining were used to eliminate false positives. The double-labeled probe Cy3-Atto-633 results in a double signal in both the red and far-red channels; autofluorescence usually appears in one channel only, and the negative control probe, labeled with Cy3, should not bind. A colon tissue with a biofilm was used as a positive control to verify correct staining with the EUB-338 probe. Bacteria were manually counted by counting the number of positive signals across the whole tissue biopsy, and bacterial density was assessed across the entire tissue section as the number of signals per mm^2^. In resection specimens, bacteria were quantified separately in the tumor or scar tissue and in adjacent non-malignant breast tissue, using ten randomly selected 1 mm² squares per region. These regions encompassed both the tumor center and the invasive tumor front. Bacterial quantification results were reported as the number of bacteria per mm². In cases with clusters of bacteria, individual signals were hard to count by eye; hence, Fiji software (version 1.53t) was used to estimate counts. Briefly, the region containing clustered bacteria was converted to a black-and-white image and imported into Fiji using appropriate scaling settings. An Otsu threshold value of 30 was applied to the binary image. To avoid edge artifacts, the image borders were excluded from the analysis. Positive white signals were then quantified using the “Analyze Particles” function, with a size threshold set from 0.02 µm² to infinity. Finally, the number of particles per µm² was calculated. In pCR cases, the tumor bed, as defined by a specialized breast pathologist, was histologically characterized by fibrotic tissue typically displaying a pattern of vessels reminiscent of granulation tissue, myxoid change, and infiltration by macrophages and/or lymphocytes.

### Bacterial DNA extraction and 16S rRNA sequencing

DNA was extracted from six 10 µm FFPE sections of tumor tissue and adjacent non-malignant tissue separately using sterile Chelex-100 in TET lysis buffer. DNA was also extracted from tissue-free paraffin regions and used as a negative control. After heating and proteinase K (Qiagen, 19133) digestion, DNA was purified via ethanol precipitation. Ice-cold 100% EtOH and 20 µl of 3 M, pH 5.2 Sodium Acetate (Merck Millipore, 1062681000) were added, followed by centrifuging at 14,000 rpm at 4 °C for 15 min. The pellet was washed twice with ice-cold 70% EtOH and spun at 14,000 rpm for 2 min at 4 °C. After air drying in the sterile fume hood for 30–45 min, 50 µl of TE buffer was added to dissolve the pellet. DNA concentration was measured with Qubit Fluorometer 2.0 (Thermo Fisher Scientific). The V3–V4 region of the 16S rRNA gene was amplified using the primers described by Klindworth et al.^32^, generating an approximately 630 bp amplicon. Amplicons were tagged with standard Illumina adapters, and uniquely indexed primers were added through a secondary PCR for each sample. PCR products were purified using Agencourt© AMPure® XP (Beckman Coulter), and DNA concentrations were determined via fluorometric analysis using Quant-iT (Invitrogen). Equimolar amounts of purified amplicons were pooled and sequenced on an Illumina MiSeq platform (paired-end 300 cycles protocol with indexing) by BaseClear BV (Leiden, The Netherlands)^32^.

### Sequence processing and microbiome data analysis

Sequencing reads were processed through a custom QIIME2 pipeline (version 23.10.1) as described previously^33,34^. Primer trimming was performed using Cutadapt (forward: 5′-CCTACGGGNGGCWGCAG-3′, reverse: 5′-GACTACHVGGGTATCTAATCC-3′) with a minimum length of 20 bp, and untrimmed reads were discarded^35^. Trimmed paired-end reads were then merged into single reads via Paired-End reAd mergeR (PEAR)^36^. Denoising was performed via DADA2 with default settings, except for the discard of forward reads with a number of expected errors higher than 6 (default 2) due to the previous PEAR step, chimera-method was set to “consensus”, and the fold-change versus the abundance of the sequence being tested was set to 2 (default 1)^37^. The output of DADA2 was imported via “qiime2 tools import”, where the biom file was constructed by biom-format as an HDF5 file format^38^, and then imported via “qiime tools import” as “BIOMV210Format”^34^. Classification of representative sequences was accomplished by “qiime2 feature-classifier classify-consensus-vsearch” via VSEARCH alignment against the SILVA-138 database (Ref NR 99; i.e., non-redundant 99% identity) as a 16S reference database for taxonomic classification^34,39^.

All analyzes were performed using R Statistical Software (v4.3.3) with OmicFlow v1.3.0 for data loading and wrangling^40^, ggplot2 v3.5.2^41^ and patchwork v1.3.2^42^ R packages for visualizations. First, the obtained rooted phylogenetic tree from “qiime2 phylogeny align-to-tree-mafft-fasttree” and table with taxonomy and their counts as BIOM format were loaded into R^34^. The data was used to select the “Bacteria” domain, and data TSS normalization (Total Sum Scaling) was performed by dividing, for each sample, the counts for each taxon by the total sum of counts for that respective sample.

Alpha diversity metrics by the Shannon index were obtained from “qiime2 diversity core-metrics-phylogenetic” with default settings^34^. PCoA with weighted UniFrac distances was computed via “qiime diversity core-metrics-phylogenetic” to visualize community structure^34^ and the vegan v2.7-1R package^43^. Metrics and relative abundance tables are provided in Supplementary data.

Amplicon Sequence Variants (ASVs) belonging to *Pseudomonas* were extracted and labeled by their respective organization. Model selection via JModelTest2 was performed to find the best model that fits the data based on Bayesian information criterion and Decision Theory (DT)^44^. Tree reconstruction is accomplished with PhyML, the K80 model with gamma distribution was selected with 100 bootstraps, other parameters were left at default settings^45^. Tree visualization was done with the ggtree v3.10.1R package^46^.

### Multiplex immunofluorescence and macrophage quantification

CD68 and CD163 were selected as markers for characterizing TAMs within the breast cancer microenvironment. CD68 is a pan-macrophage marker expressed by the majority of tissue macrophages, enabling quantification of overall macrophage infiltration. CD163 is a scavenger receptor commonly associated with alternatively activated (M2-like) macrophages, which are often linked to immunosuppressive, pro-tumorigenic functions^19^. Multiplex immunofluorescence (mIF) was performed using tyramide signal amplification (TSA) on pre-NAC biopsies and post-NAC resections using antibodies for CD163 (10D6 mouse monoclonal antibody NCL-L-CD163 1:500 (Leica Biosystems, UK), CD68 (PGM-M1 mouse monoclonal antibody M 087601 1:200 (Dako, Denmark)), and pan Cytokeratin (AE1/AE3 + 5D3 mouse multiclonal antibody 1:1000 (Abcam, Cambridge, UK)). The TSA with blue fluorophore (520 nm), red fluorophore (570 nm), and white fluorophore (780 nm) were used together with the primary antibody, respectively^47^. Slides were scanned using a Vectra 3 system (Perkin Elmer). Quantitative image analysis was conducted using QuPath v0.5.1 (https://qupath.github.io). Immune cell densities were calculated as the number of positive cells per mm², based on DAPI-guided segmentation and cell surface marker-specific classifiers. Phenotyping cell types was carried out by creating object classifiers for each marker based on the latest multiplex analysis approach available in QuPath v0.5.1. The total macrophage number was extracted (both for CD163 and CD68) and was divided by the total evaluated area to obtain the number of CD163 and CD68 macrophages per mm^2^. Analyzes were restricted to regions of interest corresponding to those evaluated by FISH. These regions were delineated on the mIF slides using the FISH annotations as a spatial reference to ensure anatomical correspondence between assays.

### Immunohistochemistry to detect *Pseudomonas*

To validate whether *Pseudomonas* detected by sequencing was due to contamination or represented true presence within breast tissue, *Pseudomonas* was visualized on resection specimens. Immunohistochemistry was performed on resection specimens using a rabbit polyclonal anti-*Pseudomonas* antibody (Abcam, ab68538, 1:1500) (see Supplementary for details).

### Statistical analysis

Data were summarized as median and interquartile range (IQR). Non-parametric tests (Wilcoxon signed-rank for paired, and Mann–Whitney U for unpaired data) were used to compare study groups. Spearman’s rank correlation was applied to assess relationships between bacterial and macrophage densities. Beta diversity comparisons were tested with PERMANOVA, on weighted UniFrac distances^48,49^. A *p*-value of <0.05 was considered statistically significant after multiple testing correction with the Bonferroni method.

## Supplementary information

Supplementary tables and figures

Supplementary data

## Data Availability

The datasets generated and/or analyzed during the current study are not publicly available due to the risk of identifying individual patients from clinical and tissue-derived data, particularly given the limited sample size, but are available from the corresponding author on reasonable request. The R scripts to reproduce the microbial analysis in this article are available in a GitHub repository: https://github.com/CMG-GUTS/Breast-Cancer-Microbiome-16S.
