# Supplementary material for: Impact of neoadjuvant chemotherapy on local breast microbiota and macrophages in ER-positive breast cancer with links to treatment response
Source: NPJ Breast Cancer. 2026 Jul 6;12:141. doi: 10.1038/s41523-026-01004-0 (PMC13720191; doi:10.1038/s41523-026-01004-0)
Supplement: Supplementary file 1 [file 41523_2026_1004_MOESM1_ESM.pdf]

Supplementary table 1 Detailed patient overview

Study code	Treatment strategy	Histological cancer type	Tumor Grade	Therapy response	Responder type	cTNM stage	(y)pTNM stage	Analysis performed
P1	neoadjuvant	Invasive carcinoma NST	1	Partial (<10% tumorrest) EUSOMA 2 (i)	Good responder	cT1mN1	ypT2N1a	FISH, DNA sequencing, mIF
P2	neoadjuvant	Invasive carcinoma NST	3	Partial (<10% tumorrest) EUSOMA 2 (i)	Good responder	cT2N1	ypT1cN1a	FISH, DNA sequencing, mIF
P3	neoadjuvant	Invasive carcinoma NST	2	Partial (10-50% tumorrest) EUSOMA 2 (ii)	Poor responder	cT2N3b	ypT1bN1M1	FISH, DNA sequencing
P4	neoadjuvant	Invasive carcinoma NST	2	Partial (10-50% tumorrest) EUSOMA 2 (ii)	Poor responder	cT2N1	ypT1cN1a	FISH, DNA sequencing, mIF
P5	neoadjuvant	Invasive carcinoma NST	2	Partial (>50% tumorrest) EUSOMA (iii)	Poor responder	cT2N0	ypT1c	FISH, DNA sequencing
P6	neoadjuvant	Invasive carcinoma NST	1	complete response, no rest tumor	Good responder	cT1mN0	ypT1bN0 (i-)	FISH, DNA sequencing, mIF
P7	neoadjuvant	Invasive carcinoma NST	2	Partial (10-50% tumorrest) EUSOMA 2 (ii)	Poor responder	cT2N0	ypT1cN0(i-)	FISH, DNA sequencing, mIF
P8	neoadjuvant	Invasive carcinoma NST	2	Partial (<10% tumorrest) EUSOMA 2 (i)	Good responder	cT2N0	ypT1cN0	FISH, DNA sequencing, mIF
P9	neoadjuvant	Invasive carcinoma NST	3	complete response, no rest tumor	Good responder	cT1N0	ypT0N0	FISH, DNA sequencing, mIF
M10	neoadjuvant	Invasive carcinoma NST	2	Partieel (<10% tumorrest) EUSOMA2 (i)	Good responder	cT4N0	ypT1cN0(i+)	FISH, DNA sequencing
M12	neoadjuvant	Invasive carcinoma NST	2	Partieel (<10% tumorrest) EUSOMA2 (i)	Good responder	cT3N0	ypT1cN0sn	FISH, DNA sequencing, mIF
M17	neoadjuvant	Invasive carcinoma NST	2	partieel (<10% tumorrest) EUSOMA2 (i)	Good responder	cT4N3c	ypT2N1mi	FISH, DNA sequencing, mIF
M18	neoadjuvant	Lobular carcinoma	2	partieel (>50% tumorrest) EUSOMA2 (iii)	Poor responder	cT2N0	ypT2mN2a	FISH, DNA sequencing, mIF
M20	neoadjuvant	Invasive carcinoma NST	2	partieel (>50% tumorrest) EUSOMA2 (iii)	Poor responder	cT1mN0	ypT2N0(i+)	FISH, DNA sequencing
M22	neoadjuvant	Invasive carcinoma NST	1	partieel (10-50% tumorrest) EUSOMA (ii)	Poor responder	cT2N0	ypT2N1a	FISH, DNA sequencing, mIF
M23	neoadjuvant	Lobular carcinoma	2	partieel (>50% tumorrest) EUSOMA2 (iii)	Poor responder	cT3N0	ypT2N0(i-)	FISH, DNA sequencing, mIF
M24	neoadjuvant	Invasive carcinoma NST	1	partieel (>50% tumorrest) EUSOMA2 (iii)	Poor responder	cT2N0	ypT2N0	FISH, DNA sequencing, mIF
M25	neoadjuvant	Invasive carcinoma NST	3	Complete pathologische respons, geen resttumor	Good responder	cT2mN1	ypT0N0(i)(sn)	FISH, DNA sequencing

M26	neoadjuvant	Invasive carcinoma NST	1	partieel (>50% tumorrest) EUSOMA2 (iii)	Poor responder	cT2N0	ypT1cN0(i-)(sn)	FISH, DNA sequencing, mIF
M27	neoadjuvant	Invasive carcinoma NST	3	Complete pathologische respons, geen resttumor	Good responder	cT2mN0	ypT0N0(i-)(sn)	FISH, DNA sequencing, mIF
M4	surgery only	Mucinous carcinoma	2	NA	NA	cT1mN0	pT2N1a	FISH, DNA sequencing
M7	surgery only	Invasive carcinoma NST	3	NA	NA	cT1N0	pT1cN0(i-)	DNA sequencing
M9	surgery only	Invasive carcinoma NST	3	NA	NA	cT1N0	pT2N0(i-)	FISH, DNA sequencing
M11	surgery only	Lobular carcinoma	3	NA	NA	cT2N0	pT2N0(i-)	FISH, DNA sequencing
M13	surgery only	Lobular carcinoma	2	NA	NA	cT1N0	pT2N1a	FISH, DNA sequencing
M14	surgery only	Invasive carcinoma NST	3	NA	NA	cT2N0	pT2N1mi	FISH, DNA sequencing
M15	surgery only	Invasive carcinoma NST	NA	NA	NA	cT1N0	pT1N0(i-)	FISH
M16	surgery only	Invasive carcinoma NST	3	NA	NA	cT1mN0	pT1cmN1a	DNA sequencing
M19	surgery only	Invasive carcinoma NST	2	NA	NA	cT1N0	pT2N0	DNA sequencing

Supplementary table 2 Clinicopathological characteristics of good and poor responders

	Good respond-ers<br>N = 10, n (%)	Poor respond-ers<br>N = 10, n (%)	p-value
Age at diagnosis in years (median, IQR)	59 [55-66]	58 [54-62]	P=0.604
Clinical tumor stage			
cT1	3 (30%)	1 (10%)	
cT2	4 (40%)	8 (80%)	P=0.971
cT3	1 (10%)	1 (10%)	
cT4	2 (20%)	0	
Pathological tumor stage			
pT0	3 (30%)	0	P=0.165
pT1	5 (50%)	5 (50%)	
pT2	2 (20%)	5 (50%)	
Pathological tumor grade (biopsy)			
Grade 1	2 (20%)	3 (30%)	
Grade 2	4 (40%)	7 (70%)	P=0.165
Grade 3	4 (40%)	0	
Tumor type			
NST	10 (100%)	8 (80%)	
Lobular	0	2 (20%)	P=0.481
Mucinous	0	0	
Missing	0	0	
PR status			
Positive	5 (50%)	6 (60%)	P=0.739
Negative	5 (50%)	4 (40%)	

Supplementary table 3 Total number of reads and reads after removing pseudomonas

Sample ID	Total number of reads	Reads belonging to pseudomonas	Total reads after removing pseudomonas
MUMC-10-H_104781_ACTGAGCGTTCTAGCT_L001	28695	27052	1643
MUMC-10-T_104780_TAGCGCTCTTCTAGCT_L001	19386	19052	334
MUMC-11T_104805_ACTGAGCGGCGTAAGA_L001	19173	18957	216
MUMC-12-H_104783_CGATCAGTTTCTAGCT_L001	28114	27450	664
MUMC-12-T_104782_CCTAAGACTTCTAGCT_L001	25199	24027	1172
MUMC-13T_104806_CCTAAGACGCGTAAGA_L001	22761	22171	590
MUMC-14T_104807_CGATCAGTGCGTAAGA_L001	28813	23366	5447
MUMC-16T_104808_TGCAGCTAGCGTAAGA_L001	19906	18235	1671
MUMC-17-H_104785_TCGACGTCTTCTAGCT_L001	32255	31982	273
MUMC-17-T_104784_TGCAGCTATTCTAGCT_L001	20474	19872	602
MUMC-18-H_104787_GGAGCTACCCTAGAGT_L001	27744	27298	446
MUMC-18-T_104786_ACTCGCTACCTAGAGT_L001	28528	28186	342
MUMC-19T_104809_TCGACGTCGCGTAAGA_L001	22875	22623	252
MUMC-20-H_104789_CGGAGCCTCCTAGAGT_L001	30355	29898	457
MUMC-20-T_104788_GCGTAGTACCTAGAGT_L001	23433	8801	14632
MUMC-22-H_104791_ATGCGCAGCCTAGAGT_L001	27061	26384	677

MUMC-22-T_104790_TACGCTGCCCTAGAGT_L001	21082	20562	520
MUMC-23-H_104793_ACTGAGCGCCTAGAGT_L001	23192	22353	839
MUMC-23-T_104792_TAGCGCTCCCTAGAGT_L001	17153	16674	479
MUMC-24-H_104795_CGATCAGTCCTAGAGT_L001	31710	30615	1095
MUMC-24-T_104794_CCTAAGACCCTAGAGT_L001	23339	22770	569
MUMC-25-H_104797_TCGACGTCCCTAGAGT_L001	31383	30967	416
MUMC-25-T_104796_TGCAGCTACCTAGAGT_L001	21629	20955	674
MUMC-26-H_104799_GGAGCTACGCGTAAGA_L001	16728	16402	326
MUMC-26-T_104798_ACTCGCTAGCGTAAGA_L001	15000	14581	419
MUMC-27-H_104801_CGGAGCCTGCGTAAGA_L001	28453	28263	190
MUMC-27-T_104800_GCGTAGTAGCGTAAGA_L001	10123	9850	273
MUMC-4-T_104802_TACGCTGCGCGTAAGA_L001	18956	18317	639
MUMC-7-T_104803_ATGCGCAGGCGTAAGA_L001	24280	13792	10488
MUMC-9T_104804_TAGCGCTCGCGTAAGA_L001	9735	8216	1519
P1-H_104763_GGAGCTACTCGACTAG_L001	20275	19884	391
P1-T_104762_ACTCGCTATCGACTAG_L001	26149	25826	323
P2-H_104765_CGGAGCCTTCGACTAG_L001	30127	29469	658
P2-T_104764_GCGTAGTATCGACTAG_L001	22335	21811	524
P3-H_104767_ATGCGCAGTCGACTAG_L001	24660	24196	464

P3-T_104766_TACGCTGCTCGACTAG_L001	24637	24144	493
P4-H_104769_ACTGAGCGTCGACTAG_L001	26286	25496	790
P4-T_104768_TAGCGCTCTCGACTAG_L001	22730	22020	710
P5-H_104771_CGATCAGTTCGACTAG_L001	29828	29197	631
P5-T_104770_CCTAAGACTCGACTAG_L001	31463	30467	996
P6-H_104773_TCGACGTCTCGACTAG_L001	33390	33065	325
P6-T_104772_TGCAGCTATCGACTAG_L001	24942	24314	628
P7-H_104775_GGAGCTACTTCTAGCT_L001	32908	32585	323
P7-T_104774_ACTCGCTATTCTAGCT_L001	24438	24000	438
P8-H_104777_CGGAGCCTTTCTAGCT_L001	20556	19636	920
P9-H_104779_ATGCGCAGTTCTAGCT_L001	28949	27900	1049
P9-T_104778_TACGCTGCTTCTAGCT_L001	18749	17516	1233

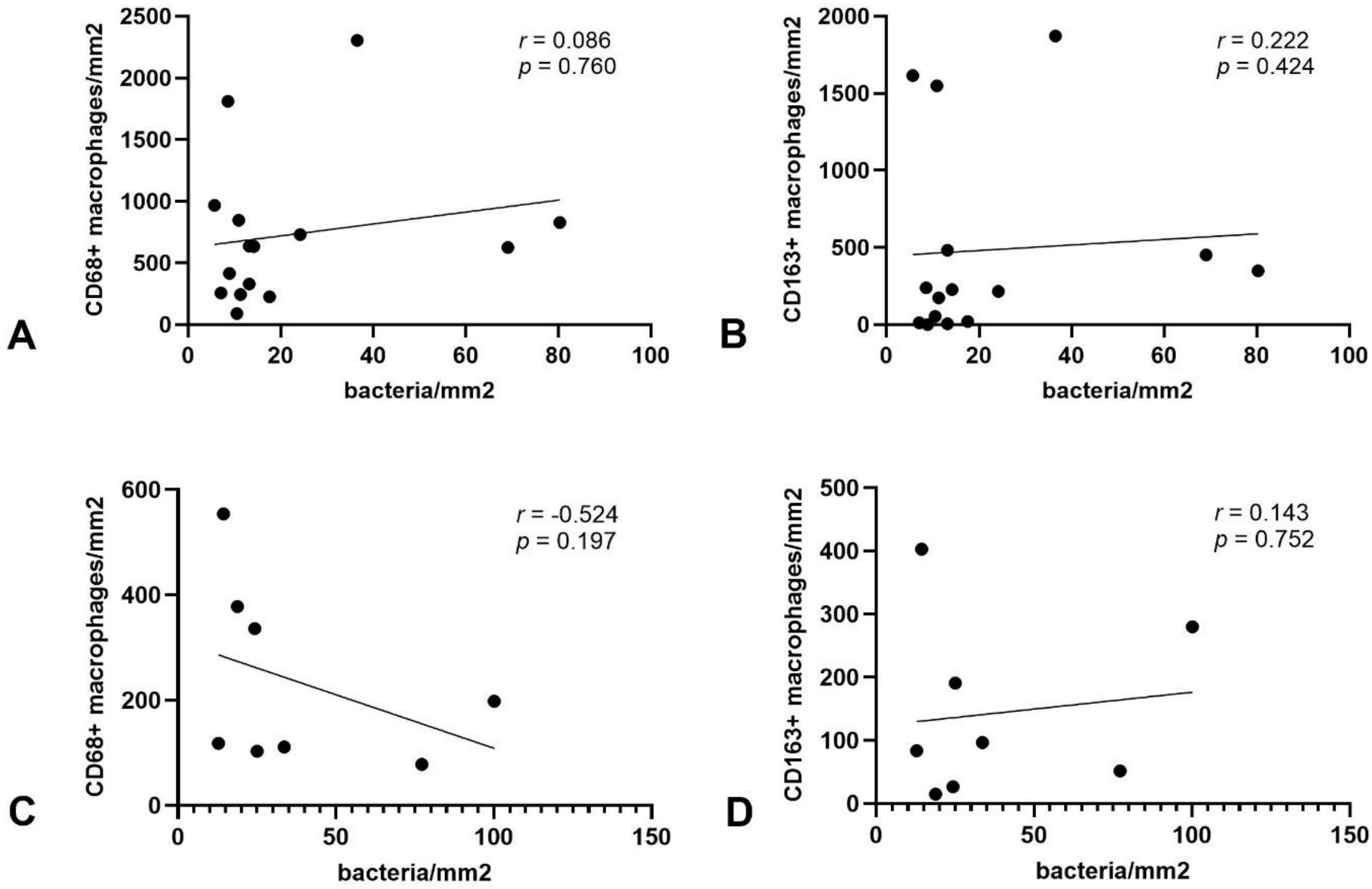

Supplementary figure 1 Spearman's correlations of bacteria and macrophages. No correlation was found between (A) preNAC CD68+ macrophages and bacteria per mm2 (r = 0.086; p = 0.760), (B) preNAC CD163+ macrophages and bacteria per mm2 (r = 0.222; p = 0.424), (C) postNAC CD68+ macrophages and bacteria per mm2 (r = -0.524; p = 0.197), and (D) postNAC CD163+ macrophages and bacteria per mm2 (r = 0.143; p = 0.752).

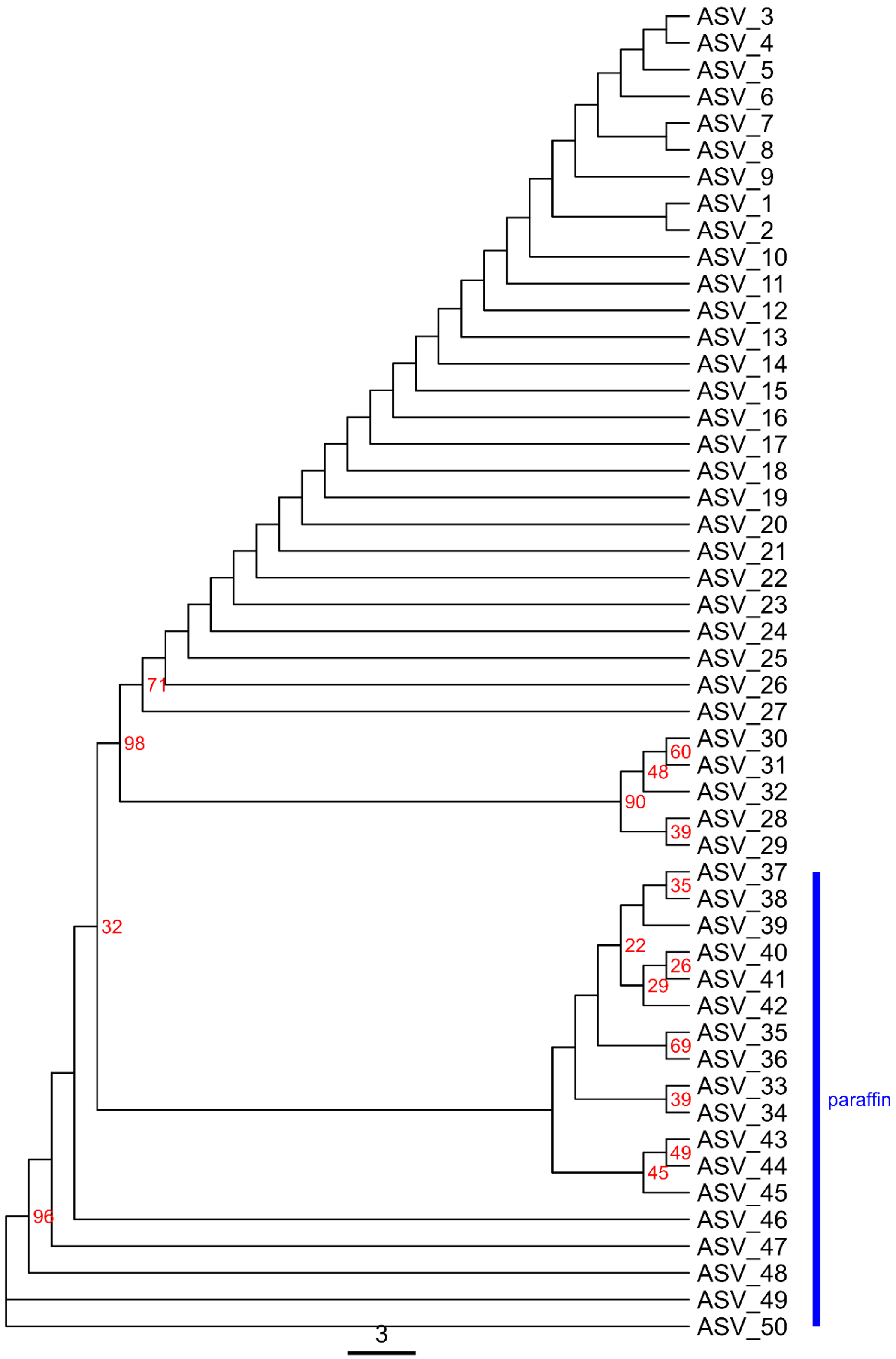

Supplementary figure 2 Phylogenetic tree reconstruction of Pseudomonas ASVs is shown. The bootstrap confidence is shown in red for each node. The paraffin controls is marked by the blue bar, which are ASV numbers 37 to 50. ASV number 27 has been found to be the most abundant and present in both PAMM and MUMC organizations. The median of the absolute abundance for MUMC is 20,000 and for PAMM is 25,000.

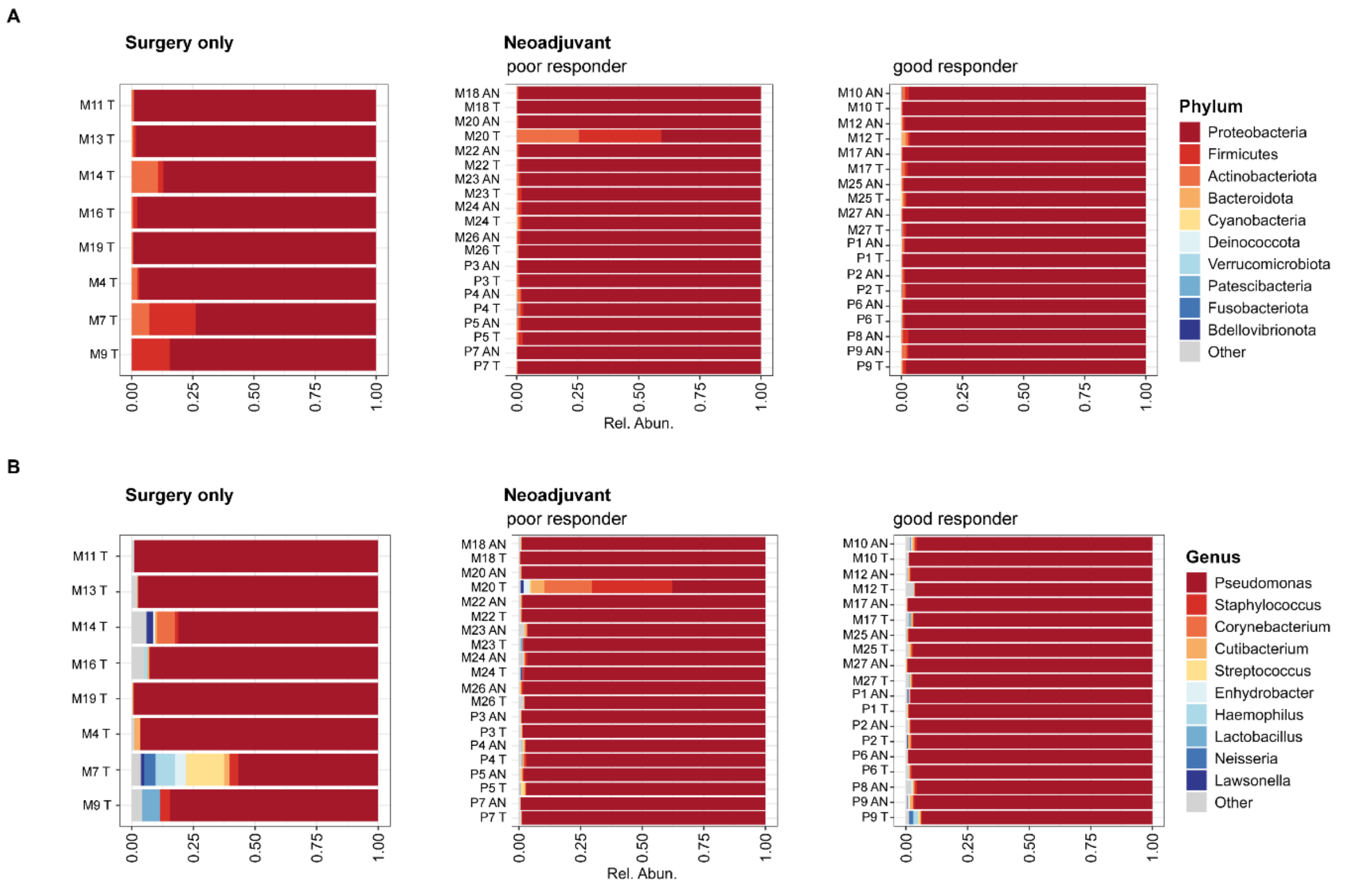

Supplementary figure 3 Overview of the relative abundance of Phyla (A) and Genera (B) for the different treatments per sample are shown, with all *Pseudomonas* amplicon sequence variants (ASVs) included.

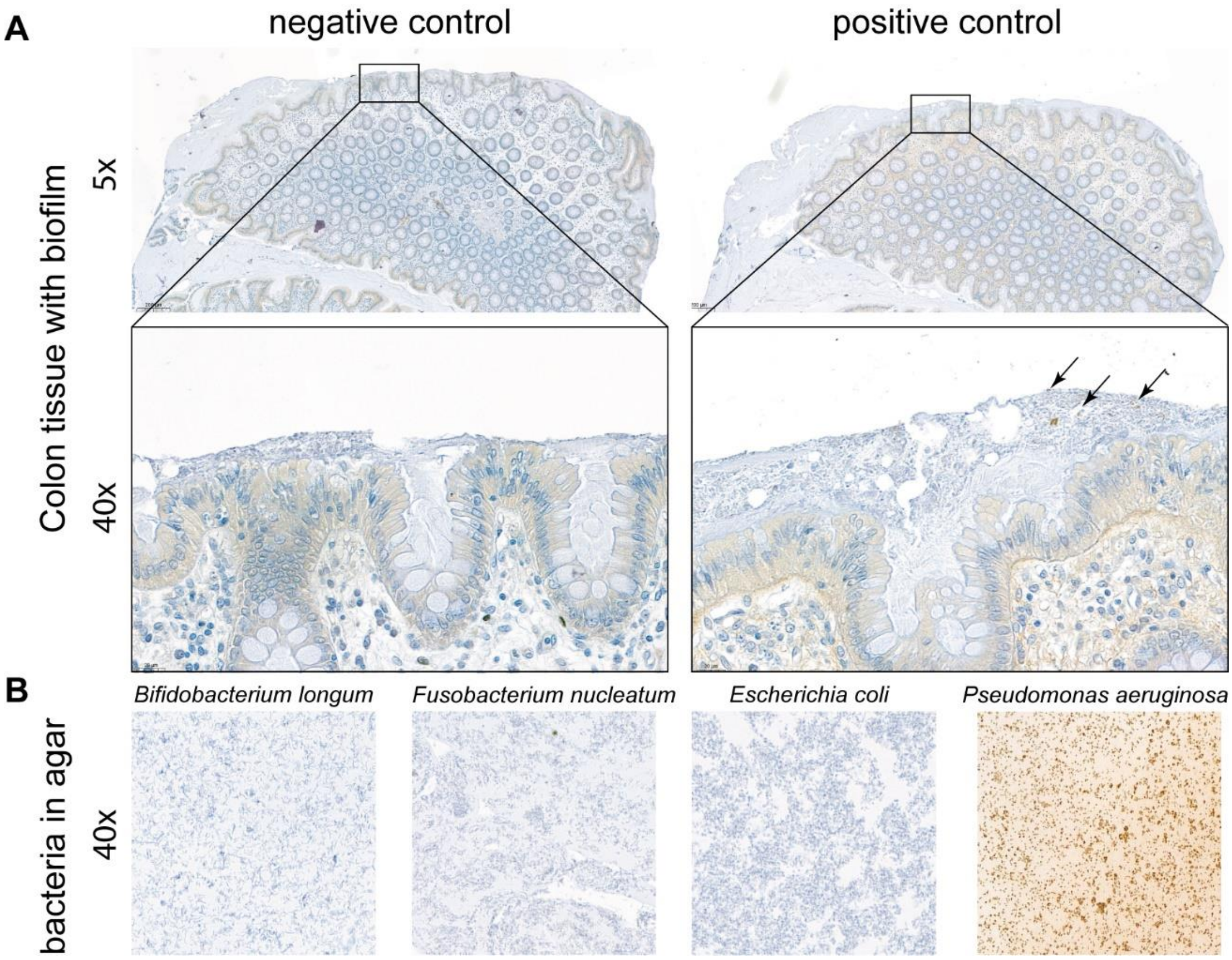

Supplementary figure 4 *Pseudomonas* IHC controls. A) *Pseudomonas* IHC without primary antibody (negative control) and with primary antibody (positive control) shows *Pseudomonas* positive bacterial cells in a biofilm positive colon biopsy (black arrows. B) The *Pseudomonas* primary antibody was tested on different bacterial strains, negative IHC is shown for *B. longum*, *F. nucleatum*, and *E. coli*, positive IHC is shown for *P. aeruginosa*.

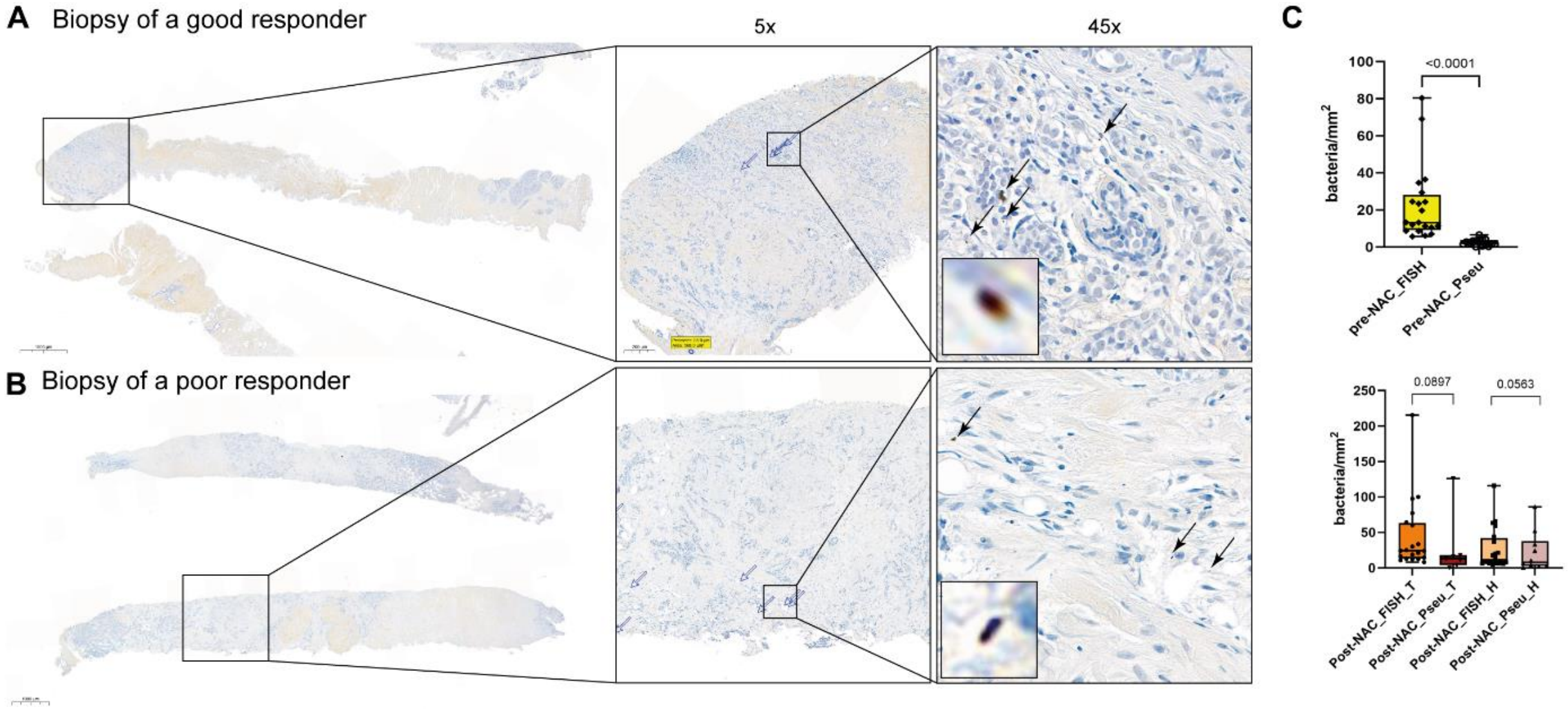

Supplementary figure 5 *Pseudomonas* detection in biopsies pre-NAC treatment Positive *Pseudomonas* detection is shown for a good responder (A) and a poor responder (B) to NAC-treatment in biopsies pre-NAC. The inlay shows a positive *Pseudomonas* signal measured in length 2.3 µm C) Quantification of bacteria per mm^2 of *Pseudomonas* IHC compared to Eubacteria FISH Pre-NAC (top-boxplot) and post-NAC (bottom-boxplot) in tumor (T) and healthy (H) areas.

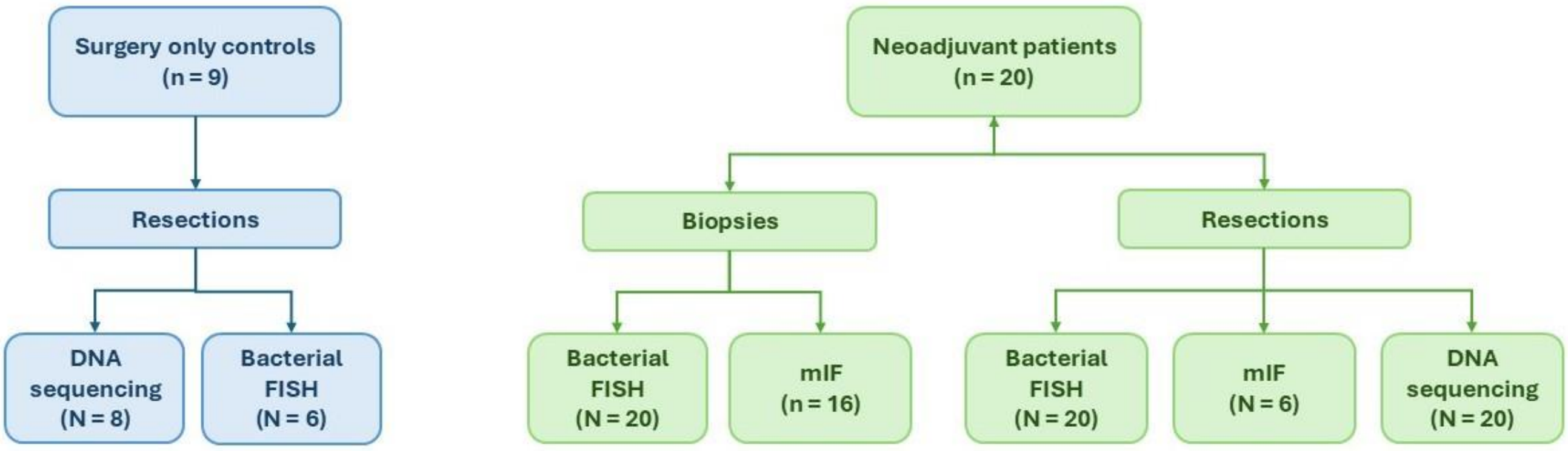

Supplementary figure 6 The flowchart summarizes the cohorts included in the study and indicates which samples were available for each downstream analysis. The surgery-only control cohort consisted of nine patients. DNA sequencing was performed in eight of these controls, while bacterial fluorescence in situ hybridization (FISH) using the EUB338 probe was performed in six controls. The neoadjuvant cohort consisted of 20 patients. Bacterial FISH was performed on all available pretreatment biopsies and matched resection specimens from these 20 patients. Multiplex immunofluorescence (mIF) was performed on pretreatment biopsies from 16 patients and on resection specimens from six patients. DNA sequencing was performed on all 20 neoadjuvant patients. Sample numbers differed between analyses because not all specimens yielded sufficient tissue of adequate quality for each technique. Fatty or fragile tissue sections were particularly susceptible to detachment during the repeated incubation and washing steps required for FISH and mIF, and in some cases tissue was washed away during processing.

Immunohistochemistry protocol for Pseudomonas detection

Tissue sections (4 µm) were mounted on Superfrost™ slides, deparaffinized, and rehydrated. Slides were then washed in TBS-T buffer, and endogenous peroxidase activity was blocked using 3% hydrogen peroxide (H_2O_2) in TBS-T. After blocking with 1% bovine serum albumin (BSA) in TBS-T for 10 minutes at room temperature, slides were incubated with a primary anti-*Pseudomonas* antibody (rabbit polyclonal, Abcam ab68538, 1:1500 dilution in TBS-T with 1% BSA) for 30 minutes at room temperature. Subsequently, slides were incubated with EnVision™ FLEX HRP for 30 minutes, and antibody binding was visualized using EnVision™ DAB. Slides were counterstained with hematoxylin and mounted using Tissue-Tek® mounting medium.
